# Seaweed-Derived Phenolic Compounds in Growth Promotion and Stress Alleviation in Plants

**DOI:** 10.3390/life12101548

**Published:** 2022-10-06

**Authors:** Omolola Aina, Olalekan Olanrewaju Bakare, Augustine Innalegwu Daniel, Arun Gokul, Denzil R. Beukes, Adewale Oluwaseun Fadaka, Marshall Keyster, Ashwil Klein

**Affiliations:** 1 Plant Omics Laboratory, Department of Biotechnology, University of the Western Cape, Robert Sobukwe Road, Bellville 7530, South Africa; 2 Department of Biochemistry, Faculty of Basic Medical Sciences, Olabisi Onabanjo University, Sagamu 121001, Ogun State, Nigeria; 3 Environmental Biotechnology Laboratory, Department of Biotechnology, University of the Western Cape, Robert Sobukwe Road, Bellville 7530, South Africa; 4 Department of Biochemistry, Federal University of Technology, P.M.B 65, Minna 920101, Niger State, Nigeria; 5 Department of Plant Sciences, Qwaqwa Campus, University of the Free State, Phuthaditjhaba 9866, South Africa; 6 School of Pharmacy, University of the Western Cape, Bellville 7535, South Africa

**Keywords:** abiotic stress, biotic stress, phenolic compounds, seaweed

## Abstract

**Simple Summary:**

This review paper discusses the importance of phenolic compounds isolated from seaweed in improving plant growth and controlling the negative effects of environmental and biological factors.

**Abstract:**

Abiotic and biotic stress factors negatively influence the growth, yield, and nutritional value of economically important food and feed crops. These climate-change-induced stress factors, together with the ever-growing human population, compromise sustainable food security for all consumers across the world. Agrochemicals are widely used to increase crop yield by improving plant growth and enhancing their tolerance to stress factors; however, there has been a shift towards natural compounds in recent years due to the detrimental effect associated with these agrochemicals on crops and the ecosystem. In view of these, the use of phenolic biostimulants as opposed to artificial fertilizers has gained significant momentum in crop production. Seaweeds are marine organisms and excellent sources of natural phenolic compounds that are useful for downstream agricultural applications such as promoting plant growth and improving resilience against various stress conditions. In this review, we highlight the different phenolic compounds present in seaweed, compare their extraction methods, and describe their downstream applications in agriculture.

## 1. Introduction

Seaweeds or macroalgae are photosynthetic multicellular organisms, found in the subtidal or intertidal part of the marine environment, free-floating or attached to surfaces such as rocks [1]. These attributes allow them to serve a variety of functions such as the provision of oxygen for the utilization of aerobic organisms, the baseline for the aquatic food chain, and the elimination of pollutants from water for other marine animals, and their relative abundance can be used to gauge the health of the marine habitat [2]. There are approximately 10,000 seaweeds and are classified based on their photosynthetic pigment as red seaweed (Rhodophyta), green seaweed (Chlorophyta), and brown seaweed (Ochrophyta) [3]. History records that seaweed has been used as far back as 300BC as a source of food [4], medicine [5], cosmetics [6], skin care [7], and agriculture [8]. Presently, seaweed has gained wide acceptance globally in various sectors such as the health food sector, pharmaceutical sector, cosmetic sector, and agriculture due to its antioxidant, anti-inflammatory, antitumoral, hypocholesterolemic, anticoagulant, antiviral, and antimicrobial properties [9]. Seaweed is used in agriculture as a bio-stimulant because a low concentration is required to induce a positive response in plant growth and increase plants’ tolerance to various stress factors [10]. However, the impact of stress on the realization of these functions is not clear because of limited research on this aspect. 

Seaweeds, especially those in the intertidal zone, are constantly being subjected to severe stress conditions such as herbivore attack, high salinity, water loss, microbial attack, and ultraviolet rays [11]. However, the negative impact of these stress conditions cannot be detected due to numerous secondary metabolites (phytochemicals) synthesized by seaweed for defense and protection [12]. Phytochemicals are compounds produced by plants essentially to offer immunity against adverse external factors but also play a vital role in plant growth and development. These phytochemicals include phenolics, alkaloids, terpenoids, saponins, glucosides, curcumins, and steroids [13]. Among the numerous phytochemicals synthesized by seaweed for survival in their habitat, phenolic compounds are the most abundant [14]. Their significant role in defense and growth regulation is due to the following properties they possess: anti-aging [15], anti-inflammation [16], antioxidant [17], antiproliferative [18], antimutagenic [19], anthelmintic [20], antigenotoxic [21], and antimicrobial effects [22]. Thus, the understanding of the stress patterns concerning the production of these phytochemicals could shed light on the mechanism of stress tolerance and the role of environmental factors in the physiology and ecology of seaweeds. 

Phenolic compounds, one of the phytochemicals produced in seaweeds, are made up of an aromatic ring with one or more hydroxyl functional groups and their structure varies from simple molecules to high-molecular-weight molecules [23]. The bioactivity of phenolic compounds is determined by the position of the hydroxy group, the number of hydroxyl groups, and the number of phenyl rings in the structure [24]. Several research works have been conducted in which phenolic compounds were isolated from seaweed and they include phlorotannin [25], flavonoids [26], phenolic acids [27], bromophenol [28], and phenolic terpenoids [29]. Seaweed-derived phenolic compounds have a wide variety of applications in the health industry for the treatment of various ailments and diseases; the food industry as preservatives and food additives; the cosmetic industry as active ingredients in cosmetics; the packaging industry to inhibit the growth of microbes; the textile industry as a source of dye; and agriculture to promote plant growth and resistance to abiotic stress factors [30]. However, there is a dearth of research on the roles of these vital compounds synthesized from seaweeds to alleviate biotic and abiotic stress in crop plants to improve agricultural productivity to meet the demands of our booming population. 

Stress in plants can be classified into abiotic and biotic and it causes physiological, morphological, and biochemical changes such as reduced rate of photosynthesis, altered gene expression, slow growth rate, and impairment in the electron transport chain [31]. Abiotic stresses are caused by environmental conditions such as drought, extreme temperatures, ultraviolet rays, and salinity, while biotic stress is caused by living organisms such as herbivores, fungi, insects, bacteria, and bacteria [32]. Stress is triggered by unfavorable external factors, which harm the growth, development, and metabolism of an organism. Abiotic and biotic stress are the major factors causing a drastic decline in crop productivity by approximately 50 percent and this continues to get worse yearly due to climatic changes and global warming [33,34]. As a result of this, there is a significant threat to food security and availability for the world’s population, which is expected to reach ten billion by 2050. A component of the United Nations 2030 Outline for Sustainable Growth is to develop agricultural practices with sustainable food production which would meet the increasing demand for food in such a way that hunger and malnutrition are controlled without an adverse effect on the environment [35]. 

Agrochemicals which include fungicides, herbicides, fertilizers, and pesticides are utilized for protecting plants against abiotic and biotic stress and to also increase crop yields [36]. Although these chemicals have increased food security, making more food available for human consumption, and thus reducing hunger, they have a negative short-term and long-term effect on animals, humans, and the ecosystem [37]. For example, organophosphorus pesticides such as glyphosate have been reported to cause various diseases such as endocrine disorders, neurological problems in infants, dementia, cardiovascular diseases, and cancer in humans [36]. There have also been several documented cases of plants developing resistance to pesticides over time. To ensure that there is an adequate food supply for the increasing human population while also protecting the environment, there is a need to utilize natural compounds such as phenolic compounds which are nontoxic, biodegradable, and as effective or better than agrochemicals [38]. 

Exogenous application of phenolic compounds can play an important role in increasing plants’ growth and mitigating the effect of abiotic and biotic stress in plants through various mechanisms such as facilitating the lignification of plant cell walls which promotes shoot length and prevents pathogens from penetrating the host plant, influencing the activity of certain enzymes such as antioxidant enzymes and the synthesis of certain compounds such as proline and phenolic compounds [39]. Singh [40] reported that there was a significant increase in the various growth parameters (shoot length, root length, total chlorophyll, and total carotenoid content) of rice seeds primed with rutin and gallic acid. Jones [41] observed that chia seedlings were better adapted to salt stress when treated with caffeic acid. In a similar study performed by Nguyen [42], it was noted that foliar application of vanillic acid and p-hydroxybenzoic acid improves the tolerance of rice seedlings to drought compared to untreated seedlings [40,41,42]. Here, we comparatively review different phenolic compound extraction methods from seaweeds and highlight the impact of these compounds towards improving plant growth under abiotic and biotic stress conditions. In addition, this review would also expand the knowledge base of plant biologists on the innovative use of seaweed-derived phenolic compounds to maximize crop yield towards sustainable food production for the ever-growing human population. 

## 2. Description and Classification of Seaweed

**Figure 1:**
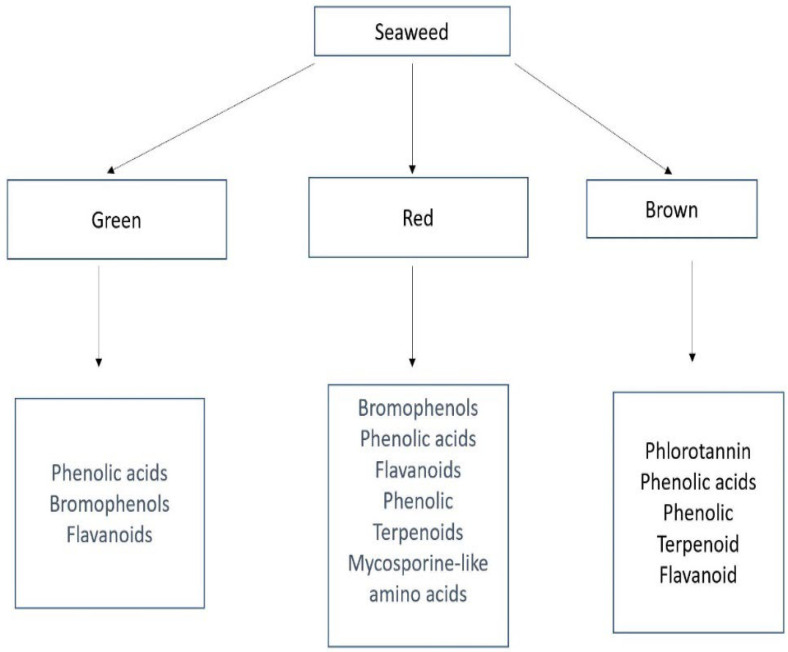
Classification of the main types of seaweed and their respective phenolic compounds.

Seaweeds, also known as macroalgae, are multicellular organisms in the marine or coastal environment that can be found attached to rocks, logs of wood, or free-floating. they perform several functions such as food, shelter, and reproductive sites for other marine animals such as sea urchins and invertebrates [3]. Their abundance, alongside other organisms, can be used as an indicator of the well-being of the marine environment. They lack true roots, stems, and leaves but have either a flexible stipe, a stronghold fast, or blades that function like a root and enable them to attach to surfaces [9]. An estimated 10,000 different types of macroalgae have been discovered. They are often categorized based on their photosynthetic pigment into three taxa (Figure 1) namely: green algae (Chlorophyta), red algae (Rhodophyta), and brown algae (Ochrophyta, class Phaeophyceae) [43].

The Chlorophyta (green algae) are widely distributed in a variety of water bodies ranging from the Arctic region, lakes, oceans, and the Antarctic. However, about 90% are reported to inhabit freshwater bodies [44]. Their size varies from microscopic ones attached to other seaweed to large macroscopic ones and they are also the foundation of the aquatic food chain. They are generally characterized as eukaryotic algae that are multicellular, oxygenic, and photosynthetic with chlorophyll (a, b) as the dominant pigment with others which are smaller such as carotenes and xanthophylls. Examples of significant green algae are *Ulva* species (sea lettuce), *Caulerpa* species, and *Chaetomorpha* species [9].

The brown seaweeds (phylum Ochrophyta, class Phaeophyceae) are the largest and most developed of the seaweeds. They are mostly found in the marine environment, especially in cold to temperate waters. Brown algae are affected the most by climatic conditions which influence their phytochemical content in different geographical zones. The presence of a pigment known as fucoxanthin contributes to its distinctive brownish color. Examples of common species of brown seaweeds are laminaria species, Ecklonia species, *Undaria* species, *Himanthalia* species, *Sargassum* species, and *Dictyota* species [45].

The phylum Rhodophyta (red seaweed) is the most abundant seaweed which has adapted to living in almost all the water bodies from fresh water, tropical, temperate, and arctic water. However, they are dominant in tropical and temperate regions. Apart from chlorophyll a, they have an additional pigment known as phycoerythrin, which makes them tolerate low light intensities. As a result of this, they can survive in deep waters and absorb light when chlorophyll “a” is no longer active [46]. Examples of common rhodophytes are coralline red seaweed, *Porphyra*, genus *Gracilaria*, and genus *Rhodymenia* [9].

## 3. Phenolic Compounds as an Important Bioactive Compound in Seaweed

The broad bioactivity of seaweed has been linked to the presence of a plethora of inorganic compounds and organic compounds which include carbohydrates, protein, lipids, vitamins, hormones, betaines, and phytochemicals (terpenoids, steroids, alkaloids, and phenolic compounds) which are more abundant than the amount found in any terrestrial plants [43]. Phenolics are among the most numerous and important bioactive compounds synthesized by seaweed, particularly brown seaweed. They are produced for protection against various abiotic and biotic stress such as ultraviolet radiation, extreme temperature, salinity, pathogenic infection, and herbivory. These phenolics also contribute immensely to the growth and development of seaweeds [24].

**Figure 2:**
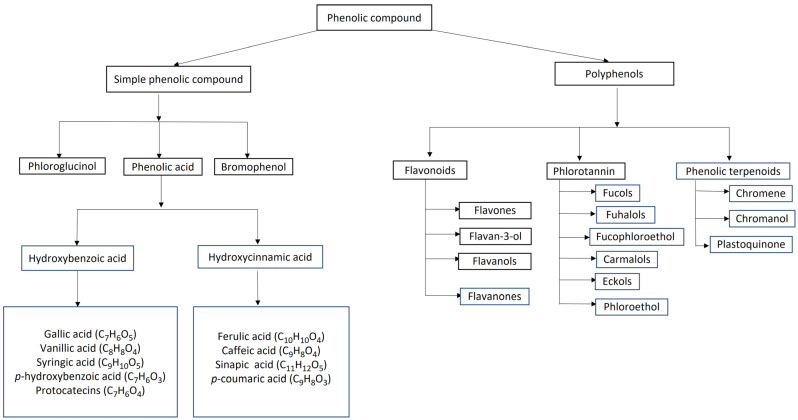
Classification of the main phenolic compounds in seaweed.

Seaweed phenolics have a basic structure of a hydroxy group attached to an aromatic ring. They are categorized by the number of carbon atoms and benzene rings in a compound as well as their solubility. Phenolic compounds with a phenol ring, such as phenolic acids and phloroglucinol, are classified as simple phenolic compounds, whereas those with multiple phenols, such as phlorotannin, are classified as polyphenols [14,24]. The different type of phenolic compounds in seaweed and the class of seaweed where they are found are further illustrated in Figure 2. 

### Extraction of Phenolic Compounds from Seaweed

Seaweeds are typically snap frozen in liquid nitrogen immediately after harvesting to halt all metabolic reactions that may result in the loss of phenolic compounds before the extraction process [47]. Following that, the seaweed samples can be extracted directly or dried before extraction. Vacuum drying, airdrying, freeze drying, or oven drying the seaweed prevents further loss of phenolic compounds and microbial growth, and ensures long-term preservation [47]. The method used to dry the seaweed has been shown to affect its phenolic content. For example, vacuum-dried *Sargassum polycystum* had the highest total phenol content compared to the other drying techniques [48].

Several methods can be used for extracting phenolic compounds from seaweed. However, due to their structural similarity, the extent of solubility, and the large molecular mass of some compounds, the chosen extraction technique should target the phenolic compound of interest. For instance, phlorotannin typically forms complexes with other metabolites within the seaweed cell walls. Therefore, an extraction method that would obtain extracts rich in phlorotannin should be adopted [45]. 

The common methods of extracting phenolic compounds from seaweed can be classified into traditional (conventional) and non-conventional (modern) methods. The conventional extraction techniques include Soxhlet extraction, maceration, and the percolation method while the nonconventional (novel) techniques include microwave-assisted extraction, enzyme-assisted extraction, ultrasound-assisted extraction, sub-critical water extraction, and subcritical CO_2_ extraction. The advantages and disadvantages of each extraction method are summarized in Table 1.

## 4. Application of Phenolic Compounds in Agriculture

### 4.1. Role of Seaweed-Derived Phenolic Compound in Promoting Plant Growth

Phenolic compounds have been used extensively to improve plant growth and increase crop yield. Data obtained from numerous studies and the literature reveal that phenolic compounds exhibit growth-promoting properties as a result of their positive impact on various phases of plant growth and developmental processes which include seed germination, shoot length, root length, plant biomass, photosynthetic pigments, and plant metabolism [57,58]. It has also been reported that the concentration used has a significant impact. While low concentrations stimulate plant growth, high concentrations tend to inhibit it. The following mechanism of action have been proposed for their growth-promoting activity: (1) by promoting cell wall formation either as precursors for lignin or by stimulating the synthesis of lignin, (2) by regulating the synthesis and breakdown of auxin in plants, (3) by stimulating leaf expansion, (4) by promoting callus growth and increasing the growth of plant roots [59].

Seed germination is a major aspect and the main determining factor of plant growth and productivity. Germinating seeds require nutrients for the growth and synthesis of the needed cellular components. These are supplied by the enzymatic hydrolysis of the food reserves such as carbohydrates, proteins, and lipids stored within the endosperm [60]. The germination process is triggered when the seeds absorb water from the environment thereby activating the synthesis of the enzymes namely α-amylase, β-amylase, catalase, protease, and peroxidase which are required for the catabolism of the food reserves into simpler molecules that can easily be absorbed by the developing seeds [61]. Phenolic compounds are known to stimulate the activity of these enzymes, thereby enhancing the rate of seed germination. In an experiment conducted by Rengasamy [59], eckol and phloroglucinol isolated from the brown algae *Eclonia maxima* were used to treat maize seeds. It was observed that there was an increased rate of germination in the treated seeds compared to the control. This was attributed to an increase in the activity of the enzyme α-amylase in the roots of eckol and phloroglucinol-treated maize seedlings, which catalyzes the breakdown of starch to simple sugars. The sugar produced was transported to the embryo to supply the needed energy for metabolism [61].

Phenolic compounds have been reported to promote the development of adventitious root and root lengthening in plants by regulating the activity of the phytohormone indole-3-acetic acid (auxin) which is the principal hormone responsible for the process. The activity of auxin is inhibited either by conjugation or decarboxylation in a reaction catalyzed by the enzyme indole-3-acetic acid oxidase (IAA oxidase). Phenolic compounds influence the root-lengthening activity of auxin by preventing the decarboxylation reaction and by acting as a cofactor that promotes the breakdown of the enzyme IAA oxidase [62]. In a recent experiment performed by Aremu [62], two types of phlorotannins, namely eckol and phloroglucinol, were isolated from *Ecklonia maxima* and their effect on *Eucomis autumnalis* was determined. It was observed that exogenous application of the isolated polyphenols caused an increase in the auxin level, which resulted in an approximately 1.5 times increase in the root length of the treated plant.

Furthermore, phenolic compounds have been shown to promote shoot lengthening by stimulating the synthesis and deposition of lignin on cell walls and cause an increase in the activity of photosynthetic pigments (chlorophyll ‘a’, chlorophyll ‘b’, total chlorophyll, and carotenoid) in plants. In an experiment performed by Rengasamy [63] on maize, polyphenols (eckol and phloroglucinol) isolated from *Ecklonia maxima* caused an increase in shoot length, root length, and photosynthetic pigment on treated maize when compared to control plants. Briand and Salamagne [64] also evaluated the effect of phlorotannin isolated from *Fucus vesiculosus* on soybean plants grown in the field. It was noted that it promoted the growth of vegetative (aerial parts) and increased pod formation, thus causing an increase in crop yield [64]. Kulkarni [65] reported that eckol increased the total chlorophyll, carotenoid, and protein content of treated spinach plant (*Spinacea oleracea* L.) which improved the crop yield and nutritional value.

### 4.2. Phenolic Compounds and Abiotic Stress Intervention in Plants

Plants, as sessile organisms, are constantly exposed to abiotic and biotic stress which disrupt plant metabolism, cause stunted growth, alter plant genetic composition, and cause a reduction in crop yield globally by approximately 50 percent and 30%, respectively [66]. Abiotic stress is environmental factors such as salinity, drought, ultraviolet light, temperature, and heavy metal accumulation, while biotic stress is the consequences of damages triggered by the action of living organisms such as bacteria, fungi, insects, nematodes, and viruses on plants [67]. These stressors have been a major concern due to their detrimental effect on plant growth and development, and it is imperative to develop an effective approach, which would enable them to withstand these adverse conditions. In the subsections below, we highlight some abiotic and biotic stresses and the phenolic compounds which have been used to alleviate their impact on plants.

#### 4.2.1. Drought

Drought is an environmental stress factor that occurs due to inadequate moisture in the soil as a result of dry weather or scarcity of surface and underground water. The effect of drought as evidenced in all the stages of plant growth and development includes a reduction in plant growth parameters (chlorophyll content, root length, shoot length, and leaf surface area), reduced germination rate, loss of cell turgor, accumulation of reactive oxygen species, and impairment of cell division which eventually reduce crop yield and availability to the population [68,69]. 

The use of phenolic acids to improve plants’ resistance to drought stress has been reported [69,70]. According to Sun [69], treating cucumber seedlings with 50µM of cinnamic acid reduced the effect of drought stress by inducing the enzymatic (catalase, ascorbate peroxidase, superoxide dismutase, and monodehydroascorbate reductase) and nonenzymatic (proline, ascorbate, reduced glutathione, and soluble sugars) defense mechanism in plants to scavenge the generated free radicals directly and indirectly, respectively. The application of vanillic acid and *p-*hydroxybenzoic acid via foliar application increased the total chlorophyll, total carotenoid content, and total antioxidant capacity thereby reversing the damaging effect of drought in treated rice plants [70]. It is worth noting that the phenolics described above have been identified and characterized in seaweed using liquid chromatography–mass spectrometry (LC-MS) [71,72]. 

#### 4.2.2. Salinity

Salinity is abiotic stress due to the buildup of salt especially sodium chloride in the soil. The common causes of salinity are human activities (bad irrigation practices, poor drainage services, and bad cultivation practices), climate change, land topography, rock weathering, and seawater deposit. Salt stress causes an accumulation of sodium and chloride ions in the leaves, as well as a decrease in the concentrations of phosphate, calcium, nitrogen, potassium, and magnesium ions. This increases the production of free radicals, disrupts ion balance, causes osmotic imbalance, and alters metabolic processes, ultimately leading to stunted growth and a massive reduction in crop yield [73]. 

Exogenous application of phenolic compounds has been shown to reverse the detrimental effects of salinity in plants and different types of phenolics have been quantified in seaweed. Babich [74] isolated vanillic acid and gallic acid from seaweed. Exogenous application of vanillic acid to salt-stressed tomato seedlings mitigates the adverse effect of salinity by influencing the antioxidant defense mechanism (enzymatic and nonenzymatic) which prevents lipid peroxidation and membrane damage; regulates the Na^+^/K^+^ balance by stimulating the absorption of potassium ion and preventing the accumulation of sodium ion; and the activity of important regulatory enzymes such as proline dehydrogenase and pyrroline-5-carboxylate synthase which increases proline synthesis and the relative water content [75].

In a study conducted by Ozfidan-Konakci [76], 0.75 and 1.5 mM of gallic acid alleviated polyethylene glycol and sodium-chloride-induced stress in three-week-old rice seedlings. It was reported that gallic acid reversed the detrimental effect of salinity by inducing the antioxidant defense mechanism (ascorbate peroxidase, catalase, superoxide dismutase, glutathione reductase, and peroxidase) which prevented the build-up of hydrogen peroxide and lipid peroxidation [76]. 

#### 4.2.3. Extreme Temperature 

Extreme temperature is a significant abiotic stress factor that influences all stages of plant development (germination, reproduction, growth, and yield) because all the metabolic processes within the plant cell are temperature-dependent. The impact of rapidly increasing temperatures on crop yields has become more frequent and intense, especially in Africa, which is expected to warm faster than the rest of the world, with an increase in the average temperature of 3–6 °C by the end of the century [77]. Although plants react to heat stress differently, the following response is common in all plants: production of reactive oxygen species, which reduces pollen viability which affects reproduction; inhibits photosynthesis; reduces seed germination; and reduces plant growth; and the denaturing of proteins, which affects enzymatic reactions in plants and reduction in crop yield [78]. 

Phenolic compounds have been shown to prevent the adverse effect of temperature extremities in plants and these compounds have been detected in seaweed.

Agregán [27] identified phenolic compounds including ferulic acid from three different brown seaweeds using liquid chromatography–diode array detection coupled to negative electrospray ionization–tandem mass spectrometry (LC-DAD–ESI-MS/MS).

According to Cheng [79], exogenous application of ferulic acid prevented the adverse effect of extreme temperature in blueberry (*Vaccinium corymbosum* L. cv. Bluecrop) seedlings. It was observed that ferulic acid treatment enhanced the transcription of genes encoding the synthesis of the antioxidant enzymes (catalase, superoxide dismutase, ascorbate peroxidase, glutathione reductase, and guaiacol peroxidase), and increased the cellular concentration of proline and soluble sugars. The increased cellular level of antioxidant enzymes prevented the accumulation of reactive oxygen species while proline and soluble sugars increased the osmotic potential and relative water content in the cell, thus preventing the detrimental effect of heat stress in the blueberry plant [79]. Exogenous application of salicylic acid reversed the effect of heat stress in ornamental pepper by activating the antioxidant defense mechanism which was evidenced by increased chlorophyll (photosynthesis rate), increased germination rate, and reduced reactive oxygen species [80].

#### 4.2.4. Heavy Metal

The effect of heavy metal stress on agricultural land due to the continuous application of fertilizers, mining, poor irrigation practices, and industrial waste is becoming a major concern globally. Recently, there has been a build-up of heavy metals such as cadmium, zinc, mercury, arsenic, lead, copper, nickel, and aluminum in the soil. Although some of these metals are needed by plants for various biochemical processes, high concentrations have the following adverse effect on plants: alteration of cell homeostasis, accumulation of reactive oxygen species, interruption of the electron transport chain, cell membrane damage, decrease in plant growth, and reduced crop yield [81]. 

Phenolic compounds, known to prevent the damages caused in plants due to heavy metal stress, have been identified in different types of seaweeds. Chakraborty [82] detected salicylic acid and other phenolic compounds such as quercetin, syringic acid, and gallic acid in *Turbinaria ornate* and *Turbinaria conoides*.

Several experimental works have been performed using phenolic compounds to mitigate the effect of heavy metal stress. For example, exogenous application of salicylic acid prevented the detrimental effect of nickel stress in mustard plants by enhancing the activities of the antioxidant enzymes and the glyoxylate enzymatic system (glyoxalase I and glyoxalase II) which improved photosynthesis and plant growth [57].

### 4.3. Phenolic Compounds and Biotic Stress Intervention in Plants

#### 4.3.1. Phenolic Compounds and Fungal Diseases

Fungal diseases are more prevalent than other biotic stressors and have a greater negative impact on crop production. Fungal infections have been linked to some of the world’s greatest famines in history. An estimated 8,000 fungi species have been identified, accounting for more than 80% of post-harvest and pre-harvest infections [83]. Fungi infect plants through the stomata; wounds; development of special organs known as appressoria, to penetrate and attach to cuticles; and by secreting hydrolytic enzymes (cellulases, cutinases, proteases, and pectinases) which enable them to invade other parts of the plant such the epidermal cell wall and cuticle. Fungal infection causes impairment of H^+^-ATPase, osmotic imbalance, decreased rate of photosynthesis, reduced crop yield, and plant death [84]. 

The use of phenolics to mitigate fungal-induced physiological damages in plants has been reported. Zhong [85] identified phenolics such as *p*-hydroxybenzoic acid and protocatechuic acid in red seaweed (*Dasya* sp., *Grateloupia* sp., and *Centroceras* sp), brown seaweed (*Sargassum* sp and *Ecklonia* sp.,), and green seaweed ( *Ulva* sp.).

These phenolics have been used to prevent early blight disease of tomato caused by *Alternaria solani* by activating the enzymatic and nonenzymatic defense mechanism, which regulates cellular homeostasis, and antioxidant balance [86]. The aforementioned compounds also enhanced the accumulation of salicylic acid within the host cell which promoted the synthesis of pathogenesis-related proteins [86]. Table 2 highlights similar examples of the antifungal effect of exogenous application of phenolic compounds.

#### 4.3.2. Phenolic Compounds and Bacteria Diseases

Plant bacterial infections are less common than fungi infections, but they are also economically significant. There are approximately 200 pathogenic bacteria species known to cause plant diseases, and they can be classified as endogenous bacteria which infect the xylem and phloem tissues, thus interrupting the transportation of water and nutrients within the plant, or exogenous bacteria, which mainly infect the intercellular spaces (apoplast) [99].

Phytopathogenic bacteria are transmitted to plants through a variety of means, including water, wind, insects, animals, and humans; however, they require openings such as stomata or wounds to penetrate the host plant. Once inside the plant, they cause diseases by synthesizing enzymes that degrade the host cell membrane and cell wall, and injecting toxins and proteins that lead to the death of the host cell [100]. 

Phenolic compounds can be used to reverse the detrimental effect of pathogenic bacteria in plants (Table 2) and these phenolics have been identified and quantified in seaweed [101]. 

According to Li [96] exogenous application of caffeic acid inhibited *Ralstonia solanacearum* infection in tobacco plants by preventing the expression of epsE and lecM genes and the formation of biofilms. In vitro, caffeic acid enhanced the activity of the enzyme phenylalanine ammonia-lyase and peroxidase, which led to the accumulation of hydroxyproline and lignin.

#### 4.3.3. Phenolic Compounds Used to Control Viral Diseases

Viral infections pose a significant challenge in agriculture due to their ability to undergo mutations and produce new variants rapidly. Furthermore, viral infections are difficult to comprehend due to the wide range of symptoms seen in host plants. Viral infections are only transmitted via vectors humans or insects, and they penetrate the host plant through wounds [102]. Viral infections influence major biochemical and physiological processes in the host plant by altering the host genetic material. Symptoms of the viral disease include wrinkling of leaves, stunted growth, phyllody, necrotic and chlorotic lesions on leaves, wilting, and the development of irregular growth patterns known as enations (galls) [103].

Apart from salicylic acid, the use of phenolic compounds to regulate plants’ resistance against viral infections remains scant. Zhang [104] showed that salicylic acid promoted plant growth and enhanced the resistance of wild soybean (*Glycine soja*) to soybean mosaic virus by stimulating the synthesis of antioxidant enzymes (catalase, peroxidase, ascorbate peroxidase, and superoxide peroxidase) and promoting the transcription of resistance-related genes (GmPR-1, GmNPR1, GmPR-10, GmEDS1, GmPR-2, and GmICS1) in the host plant. This clearly demonstrates a knowledge gap that should be addressed in future research studies.

#### 4.3.4. Phenolic Compounds Used against Herbivore and Insect Attack

Herbivores and insects pose a significant threat to plant growth and development causing approximately a 15 percent loss in crop yield annually. They further create avenues for subsequent infection by phytopathogens which increases the severity of their attack [105].

Exogenous application of gallic acid to tea plants (*Camellia sinensis*) triggered the phenyl propanoic and jasmonic acid signaling pathway which protected the plant from herbivore attack (*Ectropis obliqua* Caterpillars) by enhancing the synthesis of three antifeeding metabolites, namely epigallocatechin-3-gallate, naringenin, and naringenin [94]. These phenolic compounds have been identified and characterized from seaweed using liquid chromatography–mass spectrometry (LC-MS) [106,107]. Exogenous application of eckol stimulates the synthesis of the enzyme myrosinase which prevents the cabbage aphid (Brevicoryne brassicae) from attacking the host leaves [63]. According to a study conducted by Jan [108], kaempferol and quercetin display pesticidal effects when applied exogenously to susceptible rice strains (TN 1 strain) by reducing the vulnerability to whitebacked planthoppers by preventing the insect from feeding on the host plant and preventing egg hatching [108].

## 5. Conclusions

This review illustrates the wide range of phenolic compounds present in seaweed and highlight their agricultural importance for improved plant growth and enhanced tolerance against various abiotic and biotic stress factors. The continuous use of synthetic phenolic compounds to improve plant growth whilst minimizing the negative effects of stress conditions is no longer a viable option due to its deleterious effects on human health and the environment. The use of natural phenolic compounds derived from seaweed to improve plant growth and stress tolerance could diminish the use of synthetic chemicals thus limiting the harmful impact on the environment and improve agricultural outputs in a sustainable manner.

It is worth noting that although phenolic compounds have been identified in seaweed, their downstream application in agriculture remains limited. To date, most research has focused on the use of synthetic phenolic compounds instead of natural phenolic compounds to improve plant growth and enhance plant immunity/resilience. Therefore, more research on natural phenolic compounds is encouraged to obtain a holistic understanding of their modes of action for improved plant growth and enhanced stress tolerance especially in economically important food/feed crops.

## Figures and Tables

**Table 1:** Comparing the advantages and limitations of the extraction procedures.

Extraction Method	Advantages	Disadvantages	References
**Maceration**	Simple to operate and inexpensive	It requires the use of lots of organic solvents which makes it not eco-friendly. It is time-consuming.	[49]
**Soxhlet extraction method**	It requires the use of a smaller volume of solvent compared to other traditional extraction methods such as maceration. The solvent can be recovered and reused.	It is not eco-friendly. It causes the degradation of thermolabile compounds. Only one sample can be processed at a time.	[49]
**Microwave-assisted extraction**	It involves the use of small volumes of solvent which makes it environmentally friendly and cost-effective. It is very fast, producing a high yield of the desired phenolic compound within a short time.	It operates under high temperature and microwave power which could denature heat-sensitive compounds. It requires extra separation procedures to remove solid impurities.	[50,51]
**Ultrasound-assisted extraction**	It also involves the use of small volumes of solvent which makes it environmentally friendly and cost-effective. It is suitable for extracting thermolabile compounds because it operates at a low temperature. The equipment used is inexpensive and easily affordable compared to other nonconventional extraction techniques. It can be scaled up for industrial applications. It is very fast, producing a high yield of the desired phenolic compound within a short period.	There may be inconsistency with the distribution of sound or mechanical waves within the medium.	[24,52]
**Supercritical CO_2_ extraction**	It can be separated from the extract completely without leaving toxic remains. It is very fast and produces a high yield within a very short period. It is eco-friendly because no organic solvents are used. Carbon dioxide has a low critical temperature which makes it suitable for extracting thermolabile compounds. It can be used for small-scale and large-scale purposes. The resulting extract is devoid of inorganic salts and heavy metals because they cannot be extracted by carbon dioxide.	The equipment is highly sophisticated and expensive. It cannot be used to extract polar compounds due to the low polarity of carbon dioxide. However, polar solvents such as methanol are added in small quantities to supercritical CO_2_ to enhance their extraction.	[14,53]
**Supercritical water extraction**	It is eco-friendly because it uses water as its nontoxic solvent. It is very fast producing a high yield within a short operating time. It can be used for extracting polar compounds.	It requires the use of highly sophisticated and expensive equipment. It operates under high temperature and pressure which could denature thermolabile compounds.	[49,54]
**Enzyme-assisted extraction method**	It can be used for small-scale and large-scale production. Toxic chemicals are not utilized during the extraction process, which makes them eco-friendly. It produces a high yield of the desired phenolic compound. It can be used in conjunction with other extraction methods to obtain a higher yield.	The enzymes used could be expensive which limits their use industrially.	[55,56]

**Table 2:** Derivative phenolic compounds, uses, and their bioactivity.

Phenolic Compound	Plant Species	Type of Stress	Mechanism of Action	Reference
**Salicylic acid**	Safflower (*Carthamus tinctorius* L.).	Abiotic stress (drought)	Stimulated the nonenzymatic defense system. Increased synthesis of osmolytes. Increased synthesis of proline.	[87]
**Vanillic acid**	Blueberry (*Vaccinium corymbosum* L.)	Abiotic stress (drought)	Increased the transcription of genes encoding the synthesis of antioxidant enzymes in leaves. Increased the concentration of proline and soluble sugars. Decreased the concentration of malondialdehyde, superoxide anion, and hydrogen peroxide. Improved the relative water content.	[88]
***p*-hydroxybenzoic acid and vanillic acid**	Rice (*Oryza sativa*)	Abiotic stress (drought)	Increased the synthesis of chlorophyll “a”, “b”, carotenoids, and total phenolic compounds. Promoted plant growth rate. Enhanced the synthesis of phytoalexin momilactone (MA and MB) which increased tolerance to drought.	[70]
**Vanillic acid**	Tomato (*Solanum lycopersicum* L. cv. Pusa Ruby)	Abiotic stress (salinity)	Enhanced the glyoxalase system, thus preventing the accumulation of methylglyoxal. Activated the antioxidant defense mechanism thereby preventing lipid peroxidation and accumulation of reactive oxygen species. Increased rate of photosynthesis. Regulated the cellular Na^+^/K^+^ concentration. Improved the relative water content.	[75]
**Coumarin**	Wheat (*Triticum aestivum*)	Abiotic stress (drought)	Enhanced the activity of peroxidase, thus preventing oxidative stress. Regulated the osmotic level in the cell by regulating cellular Na^+^/K^+^ concentration. Increased synthesis of phenylalanine ammonia-lyase enzyme which increased endogenous synthesis of phenolic compound. Improved plant growth.	[89]
**Ferulic acid**	Blueberry seedlings *(Vaccinium corymbosum)*	Abiotic stress (Extreme temperature)	Enhanced the transcription of genes encoding for the synthesis of antioxidant enzymes (glutathione peroxidase and superoxide dismutase) which decreased lipid peroxidation and build-up of reactive oxygen species. Increased relative water content due to increased concentration of proline and soluble sugars.	[79]
**Salicylic acid**	*Vigna angularis*	Abiotic stress (salinity)	Increased the relative water content due to increased synthesis of glycine betaine, proline, and soluble sugar. Enhanced the enzymatic and nonenzymatic antioxidant defense mechanism. Reduction in the cellular concentration of sodium and chloride ion.	[90]
**Gallic acid**	Wheat (*Triticum aestivum* L.)	Abiotic stress (salinity)	Enhanced the activity of the antioxidant enzymes, thereby reducing reactive oxygen species and lipid peroxidation. Improved plant growth. Enhanced photosynthesis by increasing the chlorophyll content. Improved the relative water content.	[91]
**Apigenin**	Rice (*Oryza sativa L*)	Abiotic stress (salinity)	Enhanced the activity of the enzymatic (ascorbate peroxidase and catalase) and nonenzymatic defense system (endogenous flavonoids and carotenoids) thereby preventing lipid peroxidation and accumulation of reactive oxygen species. Increases the transcription of genes encoding for the synthesis of Na^+^ transporter protein, thus regulating the concentration of Na^+^/K^+^ in the cells.	[92]
**Salicylic acid**	Ornamental pepper (*Capsicum annuum* L.)	Abiotic stress (extreme temperature)	Increased chlorophyll content increased the rate of photosynthesis. Activated the enzymatic and nonenzymatic defense mechanism, thus preventing the accumulation of reactive oxygen species. Prevented degradation of cellular structures by regulating osmotic balance.	[80]
**Salicylic acid**	Mustard plant (*Brassica juncea* L.Czern. & Coss. cv. Type 59)	Abiotic stress (heavy metal)	Increased rate of photosynthesis, thus improving plant growth. Increased activity of antioxidant enzymes which prevented oxidative stress. Activated the glyoxylate system (glyoxalase I and glyoxalase II enzymes) which reduced the accumulation of toxic methylglyoxal.	[57]
**Gallic acid**	Sunflower (*Helianthus annuus*)	Abiotic stress (heavy metal)	Prevented absorption of cadmium ion by the root. Enhanced the activity of glutathione reductase, catalase, and ascorbate peroxidase which alleviated oxidative stress and increased plant growth.	[58]
**Rutin**	*Amaranthus hypochondriacus*	Abiotic stress (heavy metal)	Enhanced the synthesis of glutathione and promoted the conversion of glutathione to phytoalexins which chelate metal and prevent its accumulation within the cell. Prevents degradation of the cell membrane by inhibiting lipid peroxidation.	[93]
**Gallic acid**	Tea plant (*Camellia sinensis* cv. Longjing 43)	Biotic stress (*Ectropis obliqua* larvae)	Activated the phenylpropanoid and jasmonic acid pathway which stimulated the synthesis of metabolites such as epigallocatechin-3-gallate, naringenin, and astragalin that prevented the larvae from feeding on tea plants.	[94]
**Salicylic acid**	Green pepper (*Capsicum annuum*)	Biotic stress (antifungal)	Stimulates some immune responses in host plants such as the expression of the pathogenesis-related (PR) gene, thus inducing system resistance against the fungi. Exhibiting fungitoxic effect on the fungi and activating the synthesis of enzymes which promote the production of defense compounds.	[95]
**Eckol**	Cabbage (Brassica oleracea)	Biotic (insect repelling)	Increased the enzyme myrosinase which prevented cabbage aphid (*Brevicoryne brassicae*) from attacking the leaves.	[63]
**Caffeic acid**	Tobacco (*Nicotiana tobaccum*)	Biotic stress (antibacterial)	Increased activity of peroxidase and phenylalanine ammonia-lyase which increased the deposit of lignin in the host cell wall, thus preventing bacteria invasion. Prevented the formation of biofilm in the plant root by inhibiting the expression of *epsE and lecM genes.*	[96]
**Salicylic acid**	Pakchoi *(Brassicaceae)*	Biotic stress (antifungal)	Promoting the activity of antioxidant enzymes by increasing the expression of the respective gene. Increased concentration of proline and soluble protein which regulates the relative water content in the root and leaves cells.	[97]
***p*** **-coumaric acid**	Chinese cabbage (*Brassica rapa* var. *pekinensis*)	Biotic stress (antibacterial)	Promotes the expression of the CHS and HCT genes, thereby increasing the synthesis of endogenous phenolic compounds such as flavonoids, sinapic acid, and ferulic acid, which protects the plant from bacterial infection and promotes plant growth.	[98]

## Data Availability

Not applicable.
